# Early intervention in psychiatry through a developmental perspective

**DOI:** 10.1038/s41537-021-00137-4

**Published:** 2021-02-12

**Authors:** Michele Poletti, Andrea Raballo

**Affiliations:** 1 Child Neuropsychiatry Unit, Department of Mental Health, Azienda USL- IRCCS di Reggio Emilia, Reggio Emilia, Italy; 2 Division of Psychiatry, Clinical Psychology and Rehabilitation, Department of Medicine, University of Perugia, Perugia, Italy; 3 Center for Translational, Phenomenological and Developmental Psychopathology, Perugia University Hospital, Perugia, Italy

**Keywords:** Schizophrenia, Human behaviour

## Introduction

Adopting a developmental perspective could be a crucial maturational step to improve precision approaches in psychiatry and move the field forward in understanding the complexities of vulnerability phenotypes. Indeed, the variety of longitudinal dynamics and pathways underlying different psychopathological presentations in terms of age-at-onset and changes over time seems irreducibly articulated^1,2^. A developmental perspective in psychiatry may be helpful in the refinement of phenotypes, etiologies and trajectories^2^: this perspective is essential, particularly in those clinical conditions with established neurodevelopmental antecedents, i.e., phenotypic features already manifest in premorbid phases. Indeed, as somehow implicit in the use of the apparently neutral term of “antecedents”, it is still unclear whether these impairments generate ex novo risk for subsequent psychopathology or merely represent a temporally pristine manifestation (i.e., an early phenotypic proxy) of underlying neurobiological liability. Schizophrenia, for example, is often antedated by an interrelated constellation of childhood impairments^3–7^, as recently corroborated also by polygenic risk score (PRS) studies, confirming specific associations of schizophrenia PRS with a cascade of mutually reinforcing endophenotypic manifestations at the cognitive, social, emotional and behavioral levels since infancy through childhood to adolescence^8–11^.

A developmental approach to psychiatry^1,2^ may have significant translational implications if integrated with the current transdiagnostic clinical staging model of mental disorders^12,13^. This would definitely catalyze a developmentally-oriented understanding of illness trajectories which is essential for both personalized risk prediction and service innovation; in particular, this integration would support a better systematization of risk factors and endophenotypic manifestations that characterize premorbid and early prodromal stages (coinciding with childhood and pre-adolescence) of underlying altered neurodevelopment trajectories headed to mental disorders. Indeed, premorbid and early prodromal stages may be developmentally viewed as characterized by a progressive functional lag starting from early unspecific cognitive/motor phenotypic expressions of underling neurobiological vulnerabilities and moving towards more specific and increasing difficulties to cope with the growing demands related to age-specific developmental tasks, especially in the social domain. The usefulness of PRS in such developmental approach to early detection of risk for mental illness appears in its potential looking at specific childhood antecedents of schizophrenia, such as premorbid cognitive development, which extricate a lifelong impact.

## PRS applied to cognitive antecedents of schizophrenia

Cognitive development in premorbid/prodromal phases of schizophrenia is a complex phenomenon to investigate for several reasons^14^. First, the retrospective assessment of premorbid adjustment or estimate of premorbid IQ is intrinsically an approximation. Second, prospective studies following subjects that will develop schizophrenia since childhood (e.g., family high-risk cohorts), are few and with findings generally limited to relatively small final groups of schizophrenia patients. Finally, the investigation of cognitive antecedents of schizophrenia has to deal with the multifaceted nature of cognition (only roughly averaged by IQ) and whose multiple domains may have different developmental trajectories in subjects that will develop schizophrenia. For example, the findings of an early static deficit for verbal abilities and a later dynamic lag for executive controls, suggest a non-marginal intra and inter-individual heterogeneity across cognitive domains and developmental trajectories^15–17^.

The combination of different PRS may therefore be a useful strategy to dig into the complex puzzle of cognitive antecedents of schizophrenia. For example, a recent cluster study^18^ that investigated the intertwine between developmental cognitive trajectories, schizophrenic symptoms, global functioning and presumed genetic load, specifically indexed by PRS for schizophrenia, cognition, educational attainment, and ADHD. Clustering current and estimated premorbid IQ, three distinct trajectories of cognitive development were identified, associated with distinct clinical and functional outcomes. Those are: *stable cognitive development* (37% of the sample), weakly impacted by emerging psychosis, associated with milder schizophrenic symptoms, especially in the negative domain, and relatively benign PRS profiles (in advantage for educational attainment and in disadvantage only for schizophrenia) - *adolescent cognitive decline* (44% of the sample), associated with severe schizophrenic symptoms and global impairment, due also to a higher genetic load in terms of schizophrenia and cognition PRS in comparison with controls and cognitively stable schizophrenic patients - p*readolescent cognitive impairment* (19% of the sample), associated with intermediate schizophrenic symptoms, fewest years of education completed and low rates of employment in adulthood, due also to a generalized unfavorable genetic load, that only in this subgroup included also education and ADHD PGSs.

## Moving toward a developmentally-informed early detection approach

These findings enrich our understanding of the developmental antecedents of schizophrenia and hold substantial implications for a developmentally-sensitive approach to timely detection and risk stratification of schizophrenia spectrum conditions. Moreover, they evidence how use of multiple PRS may be better suited to disentangle the genetic architecture associated with complex phenotypic modulators, such as cognition. Cognition, indeed, although not strictly related to disease-specific symptoms, certainly exerts a prognostic influence in terms of coping and global functioning^14^. In other terms, this strongly suggests that early cognitive impairments, as well as slower cognitive growths in the transition from childhood to adolescence^17^, are important early warning signals and prognostic red-flags in case of symptomatic help-seeking or familiar risk (e.g., offspring of schizophrenic parents^4^).

In total, rather than a mere shortcut towards a diagnostic biomarker, PRS might carry an important and yet-to-be exploited broader translational potential. Indeed, although preliminary, studies on its early phenotypic expressivity in the general population could pave the way towards a deeper understanding of the neurodevelopmental pathogenesis of mental illness, as shown for schizophrenia^8–11^.

This could be implemented through a detailed analysis of (endo)phenotypic expressions and has foreseeable implications in terms of a more accurate early staging of the risk for severe mental disorders. This would be possible integrating empirical findings from child-adolescent mental health and adult mental health along an overarching developmental conceptual framework^7^.

In our opinion, a neuro-developmentally-informed clinical staging model of mental illness is essential to bridge together childhood neurobehavioral antecedents (presumably expressive of underlying genetic liability) and environmental/psychosocial triggers and reach a more comprehensive understanding of the longitudinal course of psychopathology^19^.
